# Case report of kabuki syndrome in a newborn caused by KMT2D gene mutation

**DOI:** 10.3389/fped.2024.1455609

**Published:** 2024-11-29

**Authors:** Xuejiao Ba, Xiyao Yang, Yizhi Zhang, Fang Guo, Lihong Zhu, Rui Tong, Yanbin Yang, Yuan Qian, Hongqing Zhang

**Affiliations:** ^1^ Obstetrics and Gynecology Clinical Research Center (Yunnan Joint Key Laboratory), Kunming Maternity and Child Care Hospital, Kunming, Yunnan, China; ^2^ Department of Neonatology, Kunming Maternity and Child Care Hospital, Kunming, Yunnan, China; ^3^ Department of Pediatrics, Kunming Maternity and Child Care Hospital, Kunming, Yunnan, China; ^4^ Department of Ultrasound Medicine, Kunming Maternity and Child Care Hospital, Kunming, Yunnan, China

**Keywords:** kabuki syndrome, congenital heart disease, KMT2D gene, *de novo* mutation, hyperinsulinemic hypoglycemia

## Abstract

**Background:**

Kabuki syndrome is a genetic syndrome that affects multiple organs and systems. Gene mutations are the main cause of KS. Mutations in the KMT2D and KDM6A genes have been reported as two relatively clear pathogenic pathways. This article reports a case of KS with congenital heart disease, hearing abnormalities, and hypoglycemia caused by a KMT2D gene mutation confirmed by clinical exome sequencing, enriching the clinical phenotype and gene mutation spectrum of KS and helping to improve understanding of the disease.

**Case presentation:**

Through clinical exome sequencing, we performed genetic diagnosis on a newborn with congenital heart malformation and identified a heterozygous mutation in the KMT2D gene, NM_003482.3:c.4195C>T (p.Gln1399*), which has not been reported as a pathogenic mutation before. This variant was not detected in the peripheral blood of the patient's parents, suggesting it is a *de novo* mutation.

**Discussion:**

KS has strong clinical characteristics and biological heterogeneity. Genetic diagnosis can help identify the types of mutated genes. Our results provide some clues for KS caused by KMT2D gene mutations associated with congenital heart disease, hearing abnormalities, and hypoglycemia. However, the relationship between genotype and phenotype is not yet fully understood. The molecular pathogenesis of KS still needs further exploration and clarification.

## 1 Introduction

Kabuki syndrome (KS), named for its resemblance to the makeup of traditional Japanese Kabuki theater, was initially described by Kuroki et al. (1) and Niikawa et al. (2).The KS is a rare genetic syndrome that affects multiple organs and systems. The reported incidence of KS in Japan is approximately 1 in 32,000, while in Australia, New Zealand, Europe, and America, the incidence is approximately 1 in 86,000 (3, 4), with few reports in China (5, 6).

KS type 1 and type 2 are associated with mutations in the KMT2D gene and KDM6A gene, respectively. The KMT2D gene is located on chromosome 12 and follows an autosomal dominant inheritance pattern, with pathogenic mutations present in around 75% of KS cases (7–9). The KDM6A gene is located on the X chromosome and shows X-linked dominant inheritance, with pathogenic mutations present in 3%–5% of KS cases (10–13).

In 2018, clinical and molecular genetic experts in KS developed a global consensus on diagnostic criteria for KS (14), with key clinical features including craniofacial/skeletal abnormalities, dermatoglyphic anomalies (such as persistent fingertip pads), facial dysmorphism, intellectual disability, and postnatal growth restriction (15). Despite the identification of two main causative genes, many questions regarding genetic heterogeneity, clinical phenotype heterogeneity, genotype-phenotype correlation, and underlying mechanisms remain unclear. Thus, continuous accumulation and analysis of clinical phenotype data and genetic mutation spectrum are still needed for KS patients. The prognosis of this condition is poor, and there is currently no specific treatment available. Supportive care is the main approach.

The main feature of KS is cardiac involvement. The three most common cardiac defects in KS patients are atrial septal defect (ASD), ventricular septal defect (VSD), and coarctation of the aorta (CoA) (16). Meanwhile, it has been reported that 0.3%–4% of KS patients have hyperinsulinemic hypoglycemia (HH), with the majority being associated with KDM6A-related HH (17)and fewer cases associated with KMT2D (18). HH is considered to be secondary to insulin secretion dysfunction, but the exact mechanism of its association with KS is still not fully understood. In addition, 40% of KS patients have hearing loss and 30% have middle ear dysfunction, which may be caused by immune deficiency (19), but the specific mechanism is still unclear.

This article reports a case of a newborn with a KMT2D mutation, who was found to have congenital heart defects including ASD and VSD during prenatal examination. A follow-up echocardiogram after birth revealed patent ductus arteriosus (PDA) and pulmonary hypertension (PH), and the patient experienced persistent hypoglycemia. Otoacoustic emission hearing screening (both left and right) did not pass. Clinical exome sequencing identified a novel KMT2D mutation. This report identified a rare phenotype of KMT2D gene mutation, known as HH and PDA, which expands the clinical spectrum of KMT2D gene mutations causing KS disease. The report is as follows.

## 2 Case presentation

The patient is a G1P1 male born by cesarean section at 38 + 4 weeks gestation with grade III amniotic fluid. The Apgar scores at 1/5/10 min were 8/9/9. The birth weight was 3,380 g, head circumference was 32.5 cm, and body length was 50 cm. After birth, the patient had tachypnea, irregular breathing, perioral cyanosis, groaning, nasal flaring, and tracheal tug sign. Coarse breath sounds were heard in both lungs, with coarse crackles audible.The heart rate was 180 beats per minute, regular, with normal heart sounds. A grade 2/6 systolic murmur was audible along the left sternal border, between the second and fourth ribs. Limb tone was normal and primitive reflexes were elicited, with no other abnormalities found on physical examination. The diagnosis was moderate meconium aspiration syndrome (MAS). The mother had a prenatal diagnosis of mild anemia during pregnancy. The current pregnancy was conceived naturally, with the mother being 25 years old and the father being 29 years old. They were not consanguineous and had no history of hereditary diseases.

**Figure 1:**
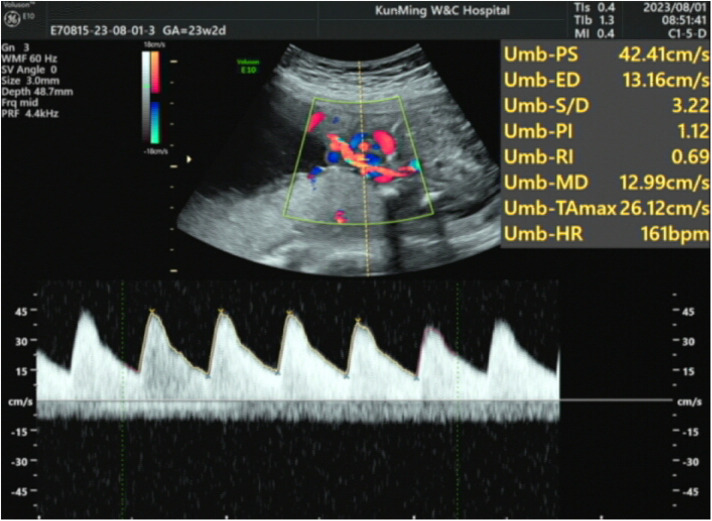
The patient underwent a fetal systemic ultrasound examination at 23 weeks and 2 days of gestation, which revealed a VSD; enlargement of the right atrium and right ventricle with mild tricuspid valve regurgitation; and narrowing of the left and right pulmonary arteries.

**Figure 2:**
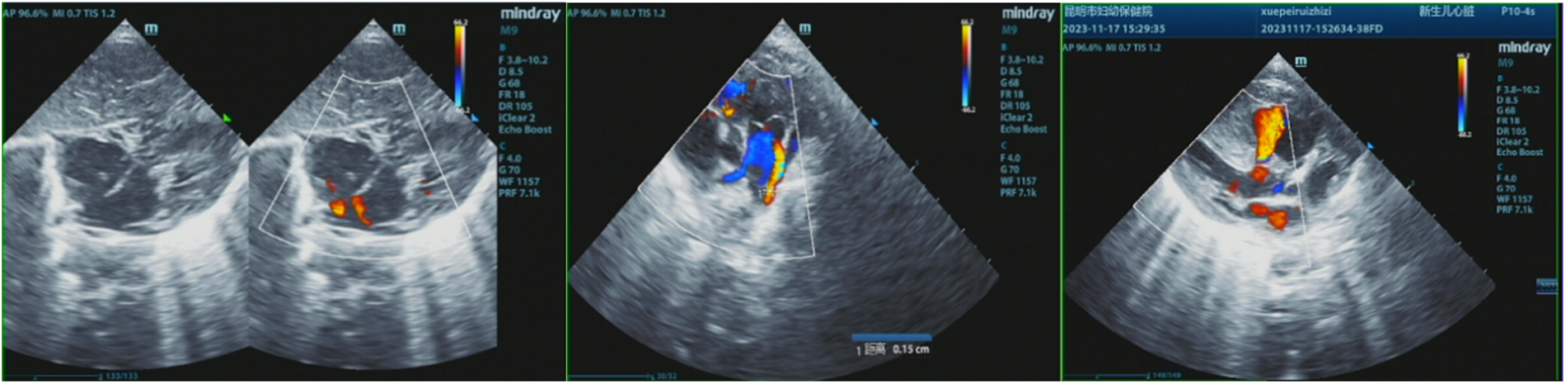
The postnatal echocardiogram results of the patient showed a VSD (6 mm, membranous) with predominant left-to-right bidirectional shunting at the ventricular level, an ASD (5 mm, central secundum) with left-to-right shunting at the atrial level, a PDA (1.5 mm) with predominant left-to-right bidirectional shunting at the arterial level, relatively narrow diameters and increased flow velocity in the bilateral pulmonary arteries, significant thickening of the interventricular septum and right ventricular anterior wall, and moderate to severe pulmonary hypertension (PH).

**Figure 3:**
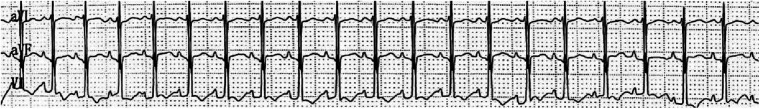
The patient's electrocardiogram showed sinus tachycardia.

The patient's mother underwent a fetal systemic ultrasound examination at 23 weeks and 2 days of gestation, which revealed the presence of a ventricular septal defect (VSD) in the fetus prenatally. There was also an enlargement of the right atrium and right ventricle, accompanied by mild tricuspid valve regurgitation, and narrowing of the left and right pulmonary arteries (Figure 1). The follow-up echocardiogram after birth showed a VSD (6 mm, membranous) with predominant left-to-right shunting at the ventricular level, an ASD (5 mm, central secundum) with left-to-right shunting at the atrial level, a PDA (1.5 mm) with predominant left-to-right shunting at the arterial level and bidirectional shunting, relatively narrow diameters and increased flow velocity in the bilateral pulmonary arteries, significant thickening of the interventricular septum and right ventricular anterior wall, and moderate to severe pulmonary hypertension (PH) (Figure 2). Electrocardiogram showed sinus tachycardia (Figure 3). Additionally, the chest x-ray revealed moderate MAS, and the patient did not pass the otoacoustic emission hearing screening test (both ears). The examination of fundus, cranial color Doppler ultrasound, abdominal ultrasound, gastric pylorus ultrasound, and electroencephalogram showed no abnormalities.

After birth, the patient developed hypoglycemia, with undetectable blood glucose levels on microanalysis and venous blood glucose levels <1.11 mmol/L. Intravenous infusion of 10% glucose, feeding, and fluid replacement normalized the blood glucose levels. The patient developed jaundice at 8 h after birth, with elevated total bilirubin, direct bilirubin, and indirect bilirubin levels. The patient also had elevated lactate dehydrogenase, creatine kinase, creatine kinase isoenzymes, and B-type natriuretic peptide levels. Electrolyte levels showed decreased sodium, calcium, and magnesium, and increased phosphorus. Infection markers such as high-sensitivity C-reactive protein, procalcitonin, and amyloid A were elevated. Other blood test parameters did not show significant abnormalities.

**Figure 4:**
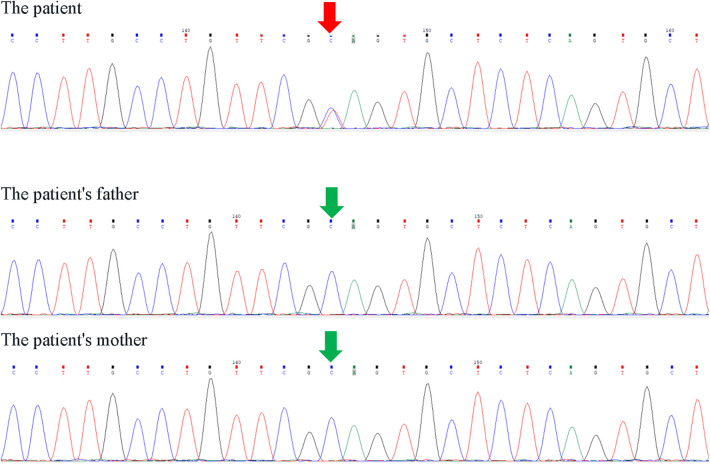
Sanger sequencing results of KMT2D gene NM_003482.3:c.4195C>T (p.Gln1399*) heterozygous mutation.

The patient underwent second-generation high-throughput clinical exome sequencing for congenital heart defects seven days after birth, and the sequencing results revealed a heterozygous mutation in the KMT2D gene, NM_003482.3:c.4195C>T(p.Gln1399*), located at position chr12:49441789 (reference GRCh37). This mutation occurred in the 15th exon of NM_003482.3 (a total of 55 exons), resulting in a premature termination of protein translation at amino acid position 1,399, truncating the protein. Furthermore, multiple functional loss variants (DM, PVSI) recorded in HGMD were found downstream of this mutation. The gnomAD database showed pLI = 1 and o/e = 0.07 (0.04–0.1) (PVS1).The variant has not yet been included in the HGMD database and gnomAD v2.1.1 (PM2_ Supporting) (Supplementary Table S1). According to the American College of Medical Genetics and Genomics (ACMG) guidelines (2015 version), this variant is classified as pathogenic. Sanger sequencing was used to confirm the KMT2D gene mutation in the patient and their parents, and it was confirmed that the parents did not carry the mutation, indicating a *de novo* mutation in the patient (Figure 4).

The patient presented with respiratory distress upon admission, with chest x-ray indicating moderate MAS. Following endotracheal intubation and mechanical ventilation, the patient's respiratory distress gradually improved. The patient was treated with digoxin and milrinone for heart failure, and furosemide was administered for diuresis to reduce cardiac load. Pulmonary artery hypertension medication was temporarily withheld. Following treatment, the patient's heart rate, blood pressure, and urine output were all normal. The patient was eventually discharged upon stabilization of the condition and diagnosed with: (1) MAS (moderate); (2) Neonatal respiratory failure; (3) Neonatal heart failure; (4) PH (moderate to severe); (5) Neonatal respiratory distress; (6) Neonatal hypoglycemia; (7) Neonatal hyperbilirubinemia; (8) Cardiovascular disorders originating from the perinatal period (ASD, VSD, PDA); (9) Neonatal pericardial effusion; (10) Neonatal hyponatremia; (11) Neonatal hypophosphatemia; (12) KS.

## 3 Discussion

In humans, the KMT2D gene is located in the chromosomal region 12q13.12 and consists of 55 exons, encoding 5,537 amino acids (20). KMT2D encodes the histone-lysine N-methyltransferase 2D protein, which is one of the six KMT proteins that function as part of a chromatin-modifying protein complex. Enhancers help regulate gene expression and often bind with transcription factors. KMT2D selectively binds to specific regions during different stages of cell differentiation to activate gene expression (21). Pathogenic/likely pathogenic variants identified in KS tend to reduce the catalytic activity of KMT2D, leading to increased levels of methylated histones and decreased expression of homologous box genes, thus affecting overall cell differentiation and potentially contributing to disease development (22). In this report, a newborn with a *de novo* mutation in KMT2D was identified. The patient underwent next-generation sequencing of clinical exomes 7 days after birth due to congenital heart defects. The sequencing results revealed a heterozygous mutation in the KMT2D gene, NM_003482.3:c.4195C>T(p.Gln1399*), with the mutation occurring at chr12:49441789 (GRCh37 reference). This variant occurs in exon 15 of NM_003482.3 (consisting of 55 exons) and results in a premature translation termination at the 1399th amino acid, causing protein truncation. Several loss-of-function variants (DM) that are recorded in HGMD downstream of this mutation were identified, with gnomAD showing pLI = 1, o/e = 0.07(0.04–0.1) (PVS1). This variant has not been recorded in the HGMD database or gnomAD v2.1.1 (PM2_Supporting) (Figure 1). According to the ACMG guidelines (2015 edition), this variant is classified as pathogenic. The KMT2D gene mutation in The patient and the parents was verified using Sanger sequencing, confirming that the parents do not carry the mutation and that The patient has a *de novo* mutation.

KMT2D can catalyze histones by methylating the fourth lysine residue in the H3 tail, enabling transcription factors on enhancers to activate transcription and thereby regulate embryonic growth, tissue differentiation, metabolism, and other processes (23, 24). KS is an autosomal dominant genetic disease caused by mutations in the KMT2D gene. These mutations can lead to defects in KMT2D and significantly affect the development of multiple organs and systems, including the craniofacial region, heart, brain, and skeleton.Migratory and nonsense mutations are considered pathogenic because they cause significant changes in protein structure, while missense mutations do not necessarily impair protein function (6). In this case report, the patient has a nonsense mutation in the KMT2D gene, which results in translation termination at the 1399th amino acid, leading to protein truncation and significant alterations in protein structure, ultimately causing the disease.

Cardiac manifestations are the main features of KS. The three most common cardiac defects in KS patients are ASD, VSD, and CoA (16), with CoA being present or absent (25). The role of KMT2D in cardiac development has been confirmed in mice. KMT2D deficiency leads to decreased expression of histone-3-lysine-4 methyltrans-ferase on cardiac enhancers and promoters, resulting in downregulation of ion transport and cell cycle genes. Furthermore, KMT2D binding regions within cardiac myocytes have been identified, and variations in KMT2D lead to reduced cardiac gene expression in knockout mice (26). One functional copy of KMT2D may be sufficient to maintain normal cardiac function, while the loss of both copies results in complete interruption of myocardial development (26). However, the exact function of KMT2D as a human cardiac regulatory gene and its role in specific congenital heart disease development are not fully understood. This report identified a patient with a novel KMT2D mutation who had congenital heart defects, including VSD and ASD, consistent with the clinical phenotype of KS heart defects reported previously (27), but the presence of PDA defect in the patient's heart was rarely reported before. Additionally, it has been reported that KS patients ultimately die from PH (28). Several factors contribute to PH, including idiopathic PH, coronary artery disease-related PH, and PH related to lung diseases, with PH patients possibly having two or more pathogenic factors (29). In this report, echocardiography of the patient with a KMT2D mutation revealed moderate to severe PH. The possible reasons for the development of PH in this patient could be attributed to the KMT2D gene mutation or lung disease. Since the patient was diagnosed with moderate MAS after birth, it is considered that the PH in this patient may be primarily related to lung disease.

Hearing abnormalities are difficult to detect clinically in infancy and depend mainly on tests such as brainstem auditory evoked potentials. In an analysis of 101 out of 462 KS patients with hearing impairment, 30 had recurrent otitis media, and the hearing loss type was unknown in 75% of cases, possibly due to immune deficiency (19). However, the specific mechanisms are still unclear. For patients with initially normal hearing, if not given attention and intervention, it is easy to further develop into conductive hearing loss or even sensorineural deafness.This report describes a newborn with a *de novo* mutation in the KMT2D gene. Both left and right ears of The patient failed the otoacoustic emissions hearing screening, suggesting possible hearing abnormalities caused by the KMT2D gene mutation. Further monitoring of The patient's hearing is needed.

HH occurs in 0.3%–4% of KS patients, which is significantly higher than the prevalence in the general population (14, 30). HH is more commonly observed in KS patients with KDM6A mutations (19), while it is less common in those with KMT2D mutations. The KDM6A gene encodes a demethylase that acts on H3K27me3/me2 and is involved in inducing stability in proliferating cells (31). Demethylation of H3K27 by KDM6A may result in dysregulation of beta cell development (32). Increased numbers of these domains in pancreatic endocrine cells lead to impaired pancreatic beta cell development and ultimately HH. However, the molecular mechanisms of HH in KS caused by KMT2D mutations remain unclear. HH is more evident in newborns, and neonatal hypoglycemia is generally defined as blood glucose levels below 40 mg/dl (2.2 mmol/L) (15). Hyperinsulinism, growth hormone deficiency, and pituitary-adrenal insufficiency may be the reasons for low blood sugar, although the specific mechanisms are not yet clear. These conditions may account for 1% of congenital hyperinsulinism cases, with most cases transitioning with glucose infusion and some requiring additional treatment with diazoxide and hormones. In this report, a newborn with the KMT2D mutation presented with persistent hypoglycemia after birth and required intravenous glucose infusion to stabilize blood sugar levels. Continuous monitoring of blood sugar and further diagnostic tests are needed to confirm whether the patient has HH and its relationship to the KMT2D mutation.

Cardiac defects are the main clinical manifestations of KS, with prenatal ultrasound examinations showing cardiac structural abnormalities in approximately 49.4% of fetuses (33), followed by hearing abnormalities in 30%–40% of KS patients, which is consistent with the clinical phenotype of cardiac defects and hearing abnormalities reported in our study. However, HH only accounts for approximately 0.3%–4% of KS cases and is mainly associated with KDM6A mutations (17), with fewer reports of KMT2D-related HH (18). This case report discovered the rare phenotypes of HH and PDA associated with KMT2D gene mutations, enriching the clinical phenotypes of KS caused by KMT2D gene mutations.

KS exhibits significant clinical heterogeneity and is prone to missed diagnosis and misdiagnosis. Moreover, due to the often insignificant facial abnormalities in newborns and varying severity of symptoms in different organs, diagnosis in newborns is particularly challenging (34). Genetic testing may help in the early diagnosis of KS. In this case report, The patient was found to have congenital heart defects in mid-pregnancy and ischemic and hypoxic symptoms after birth. Gene sequencing on the 7th day after birth revealed a heterozygous KMT2D gene mutation, suggesting a probable diagnosis of KS type 1 and providing a basis for further diagnosis and treatment. As The patient is still very young, facial features are not yet obvious, and long-term follow-up is needed to monitor facial characteristics and growth and development. This report enriches the clinical phenotype and gene mutation spectrum of KS, which helps to improve the understanding of the disease. However, the relationship between genotype and phenotype is still not fully understood, and the molecular pathogenesis of the disease still needs further exploration and clarification. Future research should expand the sample size and conduct multicenter, multi-ethnic studies to validate the findings of this case and explore the deeper relationship between KMT2D gene variations and the pathogenesis of KS. In addition, functional studies, such as studies using knock-in/knock-out animal models, will help reveal how specific mutations affect the biological characteristics of cell differentiation, development, and metabolism, and how these changes lead to the occurrence of KS.

## Data Availability

The datasets for this article are not publicly available due to concerns regarding participant/patient anonymity. Requests to access the datasets should be directed to the corresponding author.
